# Compelling Evidence for the Activity of Antiviral Peptides against SARS-CoV-2

**DOI:** 10.3390/v13050912

**Published:** 2021-05-14

**Authors:** Miray Tonk, Daniel Růžek, Andreas Vilcinskas

**Affiliations:** 1 Institute for Insect Biotechnology, Justus Liebig University of Giessen, Heinrich-Buff-Ring 26-32, 35392 Giessen, Germany; miray.tonk@agrar.uni-giesssen.de; 2 LOEWE Centre for Translational Biodiversity Genomics (LOEWE-TBG), Senckenberganlage 25, 60325 Frankfurt, Germany; 3 Department of Virology, Veterinary Research Institute, Hudcova 70, CZ-62100 Brno, Czech Republic; ruzekd@paru.cas.cz; 4 Biology Centre of the Czech Academy of Sciences, Institute of Parasitology, Branisovska 31, 37005 Ceske Budejovice, Czech Republic; 5 Department of Bioresources, Fraunhofer Institute for Molecular Biology and Applied Ecology, Ohlebergsweg 12, 35392 Giessen, Germany

**Keywords:** SARS-CoV-2, COVID-19, coronavirus, antimicrobial peptides, antiviral peptides, defensins

## Abstract

Multiple outbreaks of epidemic and pandemic viral diseases have occurred in the last 20 years, including those caused by Ebola virus, Zika virus, and severe acute respiratory syndrome coronavirus 2 (SARS-CoV-2). The emergence or re-emergence of such diseases has revealed the deficiency in our pipeline for the discovery and development of antiviral drugs. One promising solution is the extensive library of antimicrobial peptides (AMPs) produced by all eukaryotic organisms. AMPs are widely known for their activity against bacteria, but many possess additional antifungal, antiparasitic, insecticidal, anticancer, or antiviral activities. AMPs could therefore be suitable as leads for the development of new peptide-based antiviral drugs. Sixty therapeutic peptides had been approved by the end of 2018, with at least another 150 in preclinical or clinical development. Peptides undergoing clinical trials include analogs, mimetics, and natural AMPs. The advantages of AMPs include novel mechanisms of action that hinder the evolution of resistance, low molecular weight, low toxicity toward human cells but high specificity and efficacy, the latter enhanced by the optimization of AMP sequences. In this opinion article, we summarize the evidence supporting the efficacy of antiviral AMPs and discuss their potential to treat emerging viral diseases including COVID-19.

## 1. Introduction

### 1.1. SARS-CoV-2 and the Role of Antiviral Therapy Including AMPs

Severe acute respiratory syndrome coronavirus 2 (SARS-CoV-2) is a β-coronavirus that emerged in late 2019 from the Wuhan region of China [1,2,3]. SARS-CoV-2 is the agent responsible for coronavirus disease 2019 (COVID-19), which was declared a pandemic by the World Health Organization (WHO) on 11 March 2020 and has thus far caused more than 2.8 million deaths worldwide along with significant socioeconomic disruption [4,5]. Most people infected by SARS-CoV-2 are asymptomatic but still capable of transmission. Others show symptoms such as fever, cough and fatigue, ranging from mild to severe, which may last days or weeks. In the worst cases, the virus induces a cytokine storm that damages the lungs and leaves patients exposed to potentially fatal pneumonia [6,7].

Although vaccines confer protection against viruses before exposure, antiviral drugs are the first line of defense when people are already infected. However, a specific antiviral with high efficacy against SARS-CoV-2 is not yet available [8]. General antiviral drugs such as remdesivir, lopinavir/ritonavir, and umifenovir are already used, but their effectiveness is limited by the need to treat patients before the peak of viral replication [8,9]. Ribavirin may be favorable as an adjunct therapy but is not effective when administered alone [8,10]. Corticosteroids reduce mortality associated with severe COVID-19 symptoms but increase mortality in patients with coronavirus-associated pneumonia [11,12]. Intravenous immunoglobulin (IVIG) is not recommended for COVID-19 patients because there is insufficient supporting data [13]. Chloroquine and hydroxychloroquine can inhibit the replication of SARS-CoV-2 in cell lines but their clinical efficacy is unclear, especially in terms of whether the benefits outweigh the risk of dysrhythmias [14]. Interleukin-6 receptor antagonists may be beneficial in patients with cytokine release syndrome, although the effectiveness is not yet clear [15,16,17,18]. Cocktails of potent monoclonal antibodies targeting viral spike protein are effective for the treatment and prophylaxis of COVID-19, but their use is limited by the high costs of antibody production [19].

The success of antiviral therapy is dependent on many factors, including the rate of virus mutation, the genetic diversity of the viral population, the transmission route, the efficiency of viral replication, and viral persistence in the host [20]. Viruses that replicate quickly and with a greater mutation frequency can overcome antiviral drugs, so the development pipeline must be filled with new drug candidates, preferably with novel mechanisms of action [21]. There is already evidence that SARS-CoV-2 is diversifying into new lineages that are resistant to current antiviral drugs [22,23,24]. Antimicrobial peptides (AMPs) are one of the most promising categories of new antiviral drug candidates because many of them are active against viruses as well as other pathogens.

**Figure 1:**
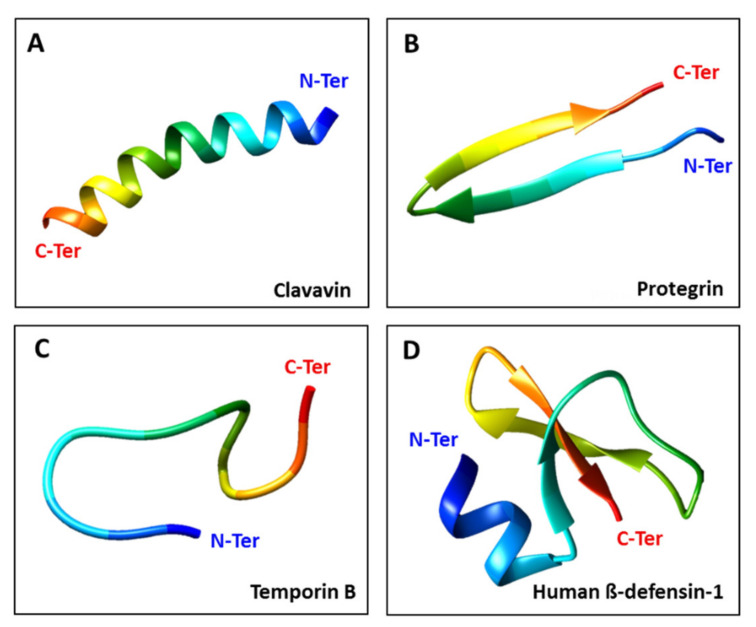
The four main structural classes of AMPs. (**A**) Clavavin adopts a typical α-helical conformation (10.2210/pdb6C41/pdb). (**B**) Protegrin PG-5 is a β-sheet peptide (10.2210/pdb2NC7/pdb). (**C**) Temporin B has a linear extension structure (10.2210/pdb6GIL/pdb). (**D**) Human β-defensin 1 features both α-helix and β-sheet structures (10.2210/pdb1IJV/pdb). The antiviral peptides were obtained from the Protein Data Bank (PDB) and adjustments were made using UCSF Chimera (http://www.cgl.ucsf.edu/chimera accessed on 22 April 2021).

AMPs are cationic peptides (net positive charge) with amphipathic characteristics (hydrophobic and hydrophilic regions) that are essential for their activity [25,26]. They can be assigned to four categories based on their structure [27]: linear α-helical peptides, β-sheet peptides, linear extension structures, and mixed α-helix and β-sheet peptides (Figure 1). Natural AMPs offer a rich source of novel antiviral agents, and additional diversity can be achieved by designing synthetic AMPs that mimic the ability of natural peptides to block critical steps in the viral life cycle [28]. The antiviral mode of action varies, but may include the direct inhibition of viral particles, competition for host cell receptors, inhibition of interactions and blocking of adsorption [29]. Some AMPs also suppress viral gene expression [30,31].

AMPs are produced naturally by all eukaryotic organisms but the analysis of AMP sequences has also facilitated the production of small molecules with similar structures (peptidomimetics) that capture the biological function of AMPs while enhancing their activity and/or reducing off-target effects [22]. AMPs offer a solution to the challenge of drug resistance because the sequences of individual AMPs can be altered very easily to keep pace with viral mutations, and two or more AMPs can be used together, or in combination with conventional treatments, to further reduce the likelihood of viral escape mutants and to exploit any additive or synergistic effects [23]. Many AMPs also have immunostimulatory properties that enhance natural innate immunity. AMPs therefore provide a new route to combination therapies involving antiviral peptides and other drugs with diverse mechanisms of action.

### 1.2. Defensins as Antiviral AMPs

Defensins are components of the innate immune system and are active against diverse pathogens [24,32]. They are cationic, amphipathic peptides, 29–42 amino acids in length, with a predominantly β-sheet structure stabilized by three disulfide bonds [32]. Their promising antiviral effects [33,34] are based on several distinct mechanisms of action, including interactions with anionic phospholipids to disrupt lipid bilayers [35] or direct binding to glycoproteins and glycolipids [36]. This aligns with the goal of researchers to find antiviral drugs that act directly against viral functions (replication, gene expression, and/or protein processing) or block viral attachment and fusion by interacting with viral proteins or their receptors on host cells [21,37,38,39].

**Figure 2:**
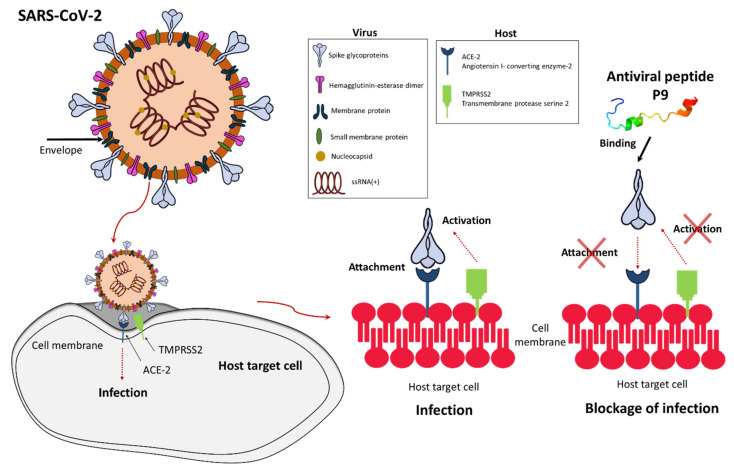
Inhibition of SARS-CoV-2 infection by antiviral peptide P9. In a normal infection, the SARS-CoV-2 spike glycoprotein binds to host cell receptor ACE-2, allowing fusion with the host cell membrane. Antiviral peptide P9 binds to the surface of the spike glycoprotein and blocks its access to ACE-2, thus preventing fusion. The secondary structure of P9 was predicted using the Phyre2 protein modeling program [51].

The first study reporting the in vitro antiviral activity of cationic AMPs involved human granulocyte defensins [40], which were shown to inhibit herpes simplex virus (HSV) types 1 and 2 and cytomegalovirus (CMV), as well as human neutrophil peptide 1 (HNP1), which inhibited vesicular stomatitis virus (VSV) [40,41]. Other defensins have been shown to confer protection against severe acute respiratory syndrome coronavirus (SARS-CoV), human immunodeficiency virus (HIV), influenza A virus, human adenovirus, human papillomavirus (HPV), HSV, and respiratory syncytial virus (RSV) [32,39,42,43]. Recently, the potent antiviral activity of defensin-like peptide P9R was demonstrated against multiple enveloped viruses that require endosomal acidification, including SARS-CoV, SARS-CoV-2, Middle East respiratory syndrome coronavirus (MERS-CoV), influenza A virus H1N1pdm09 (responsible for the 2009 pandemic of swine flu), avian influenza A virus H7N9, and also the non-enveloped rhinovirus (Table 1) [44]. P9, the parental peptide of P9R, which is derived from mouse β-defensin 4, also showed activity against SARS-CoV-2, MERS-CoV, and influenza viruses by binding to the viral glycoprotein and inhibiting endosomal acidification (Figure 2) [44,45]. Short P9 derivatives joined to the HIV Tat protein were able to deliver defective interfering influenzavirus genes that prevent endosomal acidification in vitro and in vivo [45]. Another study showed the antiviral effect of defensins against Zika virus by interacting with the surface protein and disrupting the integrity of the viral membrane [46], and against MERS-CoV by acting as a fusion inhibitor [47]. The θ-defensin retrocylin 2 (RC 2) shows activity against many viruses, including influenza virus and HSV, and a scorpion venom peptide variant (mucroporin-M1) is able to inhibit measles virus, SARS-CoV and influenza A virus H5N1 [48]. Enfuvirtide (Fuzeon or T-20) was the first antiviral peptide approved for the treatment of HIV [49] and others were approved subsequently [50]. Recently, the defensin-mimetic Brilacidin, a peptidomimetic synthetic small molecule, was shown to inhibit SARS-CoV-2 in a human lung cell line expressing ACE2 by disrupting the virus and blocking entry into cells [22]. Brilacidin together with remdesivir also showed synergistic activity against SARS-CoV-2 in vitro [22].

Several modes of action can be deployed by AMPs to inhibit the replication of SARS-CoV-2. Some AMPs exert a direct virucidal effect by targeting the viral envelope, as seen for mucroporin-M1 and brilacidin [22,48]. Others bind to the viral spike glycoprotein, thus preventing its interaction with ACE2 on host cells [52,53]. Defensin-like peptide P9R and its parental peptide P9 prevent endosomal acidification, which is necessary for uncoating during the early stages of the viral life cycle [44,54]. In addition, a natural lectin-like human intestinal defensin 5 (HD5) can shield the ACE2, preventing SARS-CoV-2 binding [55]. Finally, the indirect immunomodulatory activity of AMPs may help to reduce the severity of COVID-19 symptoms [56].

**Table 1:** Antimicrobial peptides with antiviral properties.

Antiviral Peptides	Source	Group	Sequence	Activity	Type	Mechanism of Action	Reference
P9R (β-defensin-4 derivative)	*Mus musculus*	Mouse	NGAICWGPCPTAFRQIGNCGRFRVRCCRIR	SARS-CoV-2, MERS-CoV, SARS-CoV, A(H1N1)pdm09, A(H7N9) virus, and the non-enveloped rhinovirus	RNA, Enveloped/Non-enveloped	Inhibits viral replication by binding to different viruses and then inhibits virus–host endosomal acidification to prevent the endosomal release of pH-dependent viruses	[44]
P9 (β-defensin-4)	*Mus musculus*	Mouse	NGAICWGPCPTAFRQIGNCGHFKVRCCKIR	SARS-CoV, MERS-CoV, IAV (H1N1, H3N2, H5N1, H7N7, H7N9)	RNA, Enveloped	High-affinity binding to viral glycoproteins	[54]
θ-defensin retrocylin 2	Synthetic construct	ND	GICRCICGRRICRCICGR	HIV, HSV, Influenza virus, Sindbis virus, Baculovirus	RNA, Enveloped/DNA virus	Inhibits viral fusion and entry by crosslinking membrane glycoproteins	[36,57]
MP7-NH2 (Mastoparan derivative)	*Vespula lewisii*	Insect	INLKALAALAKALL	VSV	RNA, Enveloped	Interact with the lipid component of virus membranes and thereby reduce infectivity of enveloped viruses	[58]
HEdefensin	*Haemaphysalis longicornis*	Tick	EEESEVAHLRVRRGFGCPLNQGACHRHCRSIRRRGGYCSGIIKQTCTCYRN	LGTV	RNA, Enveloped	Target virus membrane	[59]
longicin P4	*Haemaphysalis longicornis*	Tick	SIGRRGGYCAGIIKQTCTCYR	TBEV surrogate LGTV	RNA, Enveloped	ND	[60]
Mucroporin-M1	*Lychas mucronatus*	Scorpion	LFRLIKSLIKRLVSAFK	SARS-CoV, H5N1, Measles virus	RNA, Enveloped	Virus envelope interaction	[48]
Ev37	*Euscorpiops validus*	Scorpion	GLINEKKVQQYLDEKLPNGVVKGALKSLVHKAAKNQNLCAFNVDTVGMCDADCKRQGKAKGVCHGTKCKCDVELSYKK	DENV-2, HCV, ZIKV, HSV-1	RNA, Enveloped	Blocks the release of the viral genome from the endosome to the cytoplasm and then restricts viral late entry	[61]
BanLec	*Musa acuminata*	Plant	NGAIKVGAWGGNGGSAFDMG	HIV, HCP, Influenza virus	RNA, Enveloped	ND	[62]
Circulin-A	*Chassalia parviflora*	Plant	GIPCGESCVWIPCISAALGCSCKNKVCYRN	HIV	RNA, Enveloped	ND	[63]
Coccinin	*Phaseolus coccineus*	Plant	KQTENLADTY	HIV	RNA, Enveloped	HIV-1 reverse transcriptase inhibitory	[64]
Cycloviolins	*Leonia cymosa*	Plant	GVIPCGESCVFIPCISAAIGCSCKNKVCYRN	HIV	RNA, Enveloped	ND	[65]
Maculatin-1.1	*Litoria genimaculata*	Frog	GLFGVLAKVAAHVVPAIAEHF	HIV	RNA, Enveloped	ND	[66]
Caerin-1.1	*Litoria splendida*	Frog	GLLSVLGSVAKHVLPHVVPVIAEHL	HIV	RNA, Enveloped	ND	[67]
Urumin	*Hydrophylax bahuvistara*	Frog	IPLRGAFINGRWDSQCHRFSNGAIACA	H1 hemagglutinin-bearing human IAV	RNA, Enveloped	Destroys virions, targets the hemagglutinin stalk region	[68]
Tachyplesin I	*Tachypleus tridentatus*	Horseshoe crab	KWCFRVCYRGICYRRCR	VSV, IAV H1N1	RNA, Enveloped	Inactivates the VSV by destroying its envelope subunits	[69]
Cyanovirin-N	*Nostoc ellipsosporum*	Cyanobacterium	LGKFSQTCYNSAIQGSVLTSTCERTNGGYNTSSIDLNSVIENVDGSLKWQPSNFIETCRNTQLAGSSELAAECKTRAQQFVSTKINLDDHIANIDGTLKYE	HIV-1	RNA, Enveloped	Binds viral surface envelope glycoprotein gp120	[70]

SARS-CoV: severe acute respiratory syndrome coronavirus; HCV: hepatitis C virus; HSV: herpes simplex virus; VSV: vesicular stomatitis virus Indiana; HIV: human immunodeficiency virus; DENV: dengue virus type 2; ZIKV: Zika virus; IAV: influenza A virus; TBEV: tick-borne encephalitis virus; LGTV: Langat virus; H1N1: influenza A virus subtype H1N1.

### 1.3. Other Natural AMPs with Antiviral Properties

In addition to defensins, other AMPs have also demonstrated antiviral activity. The Kα2-helix peptide derived from the viral FLICE-like inhibitor protein of human gammaherspesvirus 8 (HHV-8) showed activity against influenza A virus in vitro and in vivo [71]. When Kα2 was joined to the HIV Tat peptide, the hybrid Tat-Kα2 significantly inhibited influenza A virus replication and transmission, and protected mice challenged with lethal doses of the highly pathogenic H5N1 and H1N1 strains [71]. The Tat-Kα2 peptide was shown to destabilize viral membranes, depending on the lipid composition of the viral envelope [59]. Kα2 also inhibited infections with enveloped viruses such as VSV and RSV but with negligible cytotoxicity [59]. The antiviral peptide urumin from a south Indian frog was also active against influenza A virus strains that are resistant to oseltamivir, zanamivir, and peramivir [68].

The AMP database DRAMP currently lists 2015 antiviral AMPs from diverse origins [62], which will facilitate the identification of antiviral candidates that inhibit the virus infection cycle either by directly blocking functions such as replication or by obstructing virus–host interactions or trafficking. In the latter case, antiviral peptides that block the endosomal acidification of pH-dependent viruses are promising because this includes many of the highly pathogenic viruses that have emerged in recent years [68].

### 1.4. Advantages and Disadvantages of AMPs

Antiviral drug resistance is a public health concern because resistant mutants can spread rapidly to become predominant in the population [72,73]. The emergence of avian influenza A(H1N1)pdm09 mutants resistant to the AMP P9R and the approved antiviral drug zanamivir has been compared, revealing that resistance to zanamivir emerged after 10 passages in the presence of the drug whereas no resistance to P9R was observed even after 40 passages [44].

Other advantages of AMPs include the ability to produce new AMPs rapidly, and the high therapeutic ratio of many AMPs, combining high efficacy with low toxicity in humans [74,75,76]. It is therefore possible to achieve high specificity and effectiveness at low concentrations with minimal side effects [77,78,79,80]. For example, the Zika virus inhibitor Z2 showed low toxicity in vitro and in vivo in pregnant mice and fetuses, which suggests the drugs could be safe for use by pregnant women [46].

Conversely, the drawbacks of AMPs include their short half-life and high costs of production [27,29,81]. The former can be addressed by using amino acid d-enantiomers, which are less susceptible to proteolytic enzymes while maintaining antiviral activity [82]. Cost reductions can be achieved by optimizing the AMP synthesis and purification methods, including the replacement of chemical synthesis with the production of recombinant peptides in host cells that are not susceptible to the specific AMP product [83].

AMPs have low immunogenicity [84] and they can be also used in combination with other drugs to enhance their efficacy at lower concentrations [27]. Cocktail therapies based on combinations of natural and/or synthetic AMPs or other antiviral agents could also be tested for the treatment of COVID-19 [56]. However, more studies are needed to confirm the effectiveness and safety of such approaches, particularly in the case of pregnant women and immunocompromised patients.

## 2. Conclusions

Emerging and re-emerging viruses have caused severe socioeconomic disruption over the last two decades, reminding us that we are still unprepared to deal with viral pandemics. This reflects the lack of effective antivirals, removing an essential front line treatment option and making us reliant on vaccination to avoid significant morbidity and mortality. In this opinion article, we have highlighted the potential of AMPs as candidate antiviral drugs or drug leads. AMPs can be produced rapidly by chemical synthesis or the expression of recombinant peptides, and can be modified to improve their efficacy and stability *in vivo*. AMPs could therefore be used in the fight against COVID-19 and future viral pandemics either as new first-line treatments or as adjuncts to existing antiviral drugs. Given the broad and potent activities of AMPs against multiple viruses, we recommend the funding of research into the development of AMPs as a new therapeutic strategy against viral diseases.
